# Organo-Mediated Ring-Opening Polymerization of Ethylene Brassylate from Cellulose Nanofibrils in Reactive Extrusion

**DOI:** 10.1021/acssuschemeng.4c01309

**Published:** 2024-07-12

**Authors:** Angelica Avella, Abdolrahim Rafi, Luca Deiana, Rosica Mincheva, Armando Córdova, Giada Lo Re

**Affiliations:** † Department of Industrial and Materials Science, Chalmers University of Technology, Rännvägen 2A, Göteborg 41258, Sweden; ‡ Department of Natural Sciences, Mid Sweden University, Holmgatan 10, Sundsvall 85170, Sweden; § Laboratory of Polymeric and Composite Materials (LPCM), Center of Innovation and Research in Materials and Polymers (CIRMAP), University of Mons, Mons 7000, Belgium

**Keywords:** reactive extrusion, ethylene brassylate, cellulose nanofibrils, ring-opening polymerization, grafting, organic catalyst

## Abstract

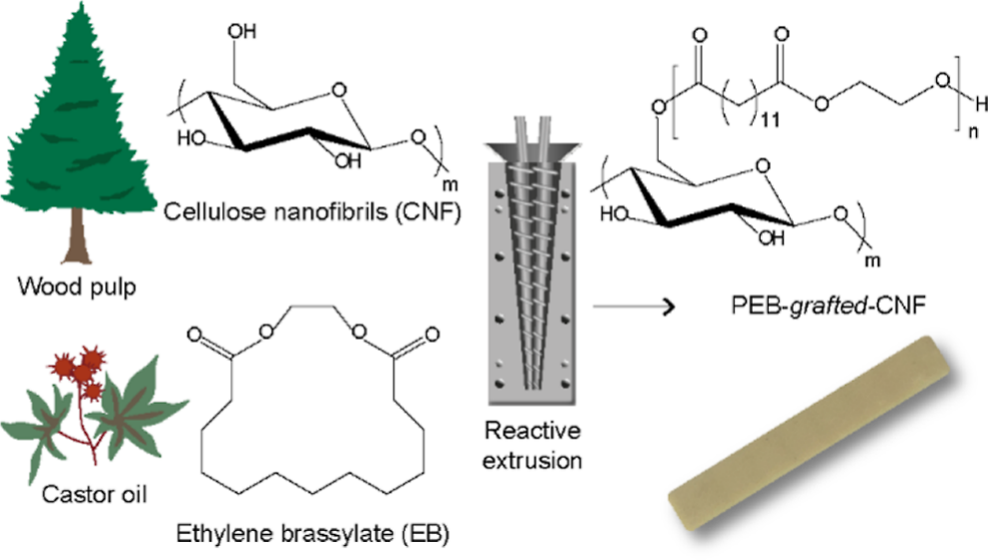

Ethylene brassylate is a renewable macrolactone from castor oil that can be polymerized via ring-opening polymerization (ROP) to obtain a fully biosourced biodegradable polyester. ROP mediated by organometallic catalysts leads to high molar mass poly(ethylene brassylate) (PEB). However, the use of metal-free organocatalysis has several advantages, such as the reduction of toxic and expensive metals. In this work, a novel cellulose nanofibril (CNF)/PEB nanocomposite fabrication process by organocatalysis and reactive extrusion (REx) is disclosed. Here, ROP was carried out via solvent-free REx in the presence of CNFs using organic 1,5,7-triazabicyclo[4.4.0]dec-5-ene as a catalyst. Neat or lactate-esterified CNFs (LACNF) were used as initiators to investigate the effect of surface topochemistry on the in situ polymerization and the properties of the nanocomposites. A molar mass of 9 kDa was achieved in the presence of both unmodified and LACNFs with high monomer conversion (>98%) after 30 min reaction in a microcompounder at 130 °C. Tensile analysis showed that both nanofibril types reinforce the matrix and increase its elasticity due to the efficient dispersion obtained through the grafting from polymerization achieved during the REx. Mechanical recycling of the neat polymer and the nanocomposites was proven as a circular solution for the materials’ end-of-life and showed that lactate moieties induced some degradation.

## Abstract 2

Ethylene brassylate was polymerized in situ onto cellulose nanofibrils to produce biosourced and biodegradable composites via reactive extrusion.

## Introduction

Biobased, recyclable, and biodegradable polymers are needed to fulfill the sustainable goals of limiting fossil fuel dependence and reducing the environmental impact of plastics after their use. The requirements of renewability and biodegradability are simultaneously met by a few commercial polymers such as polylactide, polyhydroxyalkanoates, and starch blends.^1^ However, the recyclability of such polyesters is still a challenge. Ethylene brassylate (EB) is a relatively cheap and commercially available renewable macrolactone that is derived from castor oil and can serve as a monomer for the synthesis of a biodegradable aliphatic polyester. Ring-opening polymerization (ROP) of EB has been carried out at a lab scale through enzymatic,^2^ organometallic,^3−9^ or organic^4,10−12^ catalysis. Not commercially available yet high molar mass poly(ethylene brassylate) (PEB) (>200 kDa) can achieve similar properties to commercial fossil-based poly(ε-caprolactone) via organometallic catalysis.^3,4^

Compared to metallic ones, organic catalysts have demonstrated reduced toxicity and environmental impact, relatively lower price than enzymatic counterparts, and they are suitable for the synthesis of polymers for biomedical and electronic applications.^13,14^ The use of organic catalysts for EB polymerization was first reported by Pascual et al.^10^ who demonstrated that the strong base 1,5,7-triazabicyclo[4.4.0]dec-5-ene (TBD) was the most efficient catalyst among the ones tested. Fernández et al.^4^ compared EB polymerization mediated by triphenyl bismuth (Ph_3_Bi) versus TBD. They discovered that TBD-catalyzed PEB shows higher glass transition temperature (*T*_g_) and is stiffer, more crystalline, and more thermostable than Ph_3_Bi-catalyzed PEB, despite the lower molar mass (32 kDa). Some studies have explored the dual role of TBD as both a catalyst and an initiator.^4,11,12^ In particular, Pascual et al.^11^ polymerized EB with or without benzyl alcohol (BnOH) as an initiator, and higher molar masses were achieved without alcohol, as TBD competes with BnOH for the initiation.

To our knowledge, in all previous studies, the ROP of EB has been carried out in solution or bulk, both discontinuous (batch) conditions. This work aimed to assess the feasibility of polymerizing EB via reactive extrusion (REx) in conventional melt processing equipment on a 15 cm^3^ scale under controlled conditions. This technology eliminates the need for organic solvents, and if optimized, it can provide a scalable continuous method for EB polymerization and manufacturing in a single processing step. REx also increases the monomer diffusion during the polymerization, often hindered in bulk due to higher polymer viscosity,^10,11^ thus leading to higher conversion and faster kinetics.^15^

To improve polymer matrices, nanofillers can be employed to produce nanocomposites with larger thermomechanical properties, even at low loadings (<5 wt %). Among the nanofillers, nanocellulose is a biobased and biodegradable material which in the nanofibril form (CNF) is characterized by a high aspect ratio (80–300), high stiffness (≈100 GPa), and relatively low density (≈1.5 g cm^–3^).^16^ One study has reported the fabrication of PEB nanocomposites with cellulose nanocrystals (CNC).^3^ In this case, EB was polymerized by catalysis with Ph_3_Bi, and it was blended in a second step in chloroform solution with CNC up to 50 wt %. Both deformation and tensile strength improved with CNC up to 5 wt %; however, agglomeration occurred for contents above 2.5 wt %. The hydroxyl groups on nanocellulose surfaces form strong intra- and internanocellulose interactions, challenging their dispersion into organic solvents and in the relatively more hydrophobic polymer matrices. Poor dispersion is correlated to low reinforcement efficiency, as it reduces the surface contact between the nanocellulose and the polymer, thus limiting the stress transfer between the phases. Moreover, the nanocellulose agglomerates in the polymer melt, resulting in debonding and pull-out phenomena observed in the morphology of solid composites.

One strategy to improve the CNF dispersion and interaction with polymer matrices is grafting polymeric chains onto or from the cellulose surfaces.^17^ In previous work, we have successfully used cellulose as an initiator for organic acid-catalyzed ROP of ε-caprolactone and lactide.^18−20^ However, the tartaric acid failed to catalyze the ROP of EB under neat conditions.^18,20^ Herein, we disclose a novel semicontinuous organocatalyzed synthesis of recyclable PEB containing CNF. The aim is to polymerize EB in situ and simultaneously manufacture nanocomposites in a single-step process. The polymerization of EB was carried out during REx in the presence of CNF to facilitate their dispersion and possibly initiate the ROP from the CNF hydroxyl groups. Lactate-esterified CNF (LACNF) was also used to evaluate the effect of different topochemical moieties on both the polymerization and the nanocomposites’ properties. Moreover, to evaluate the recyclability as an end-of-life strategy, we assessed the circularity of PEB and the nanocomposites by mechanical recycling via extrusion.

## Experimental Section

### Materials

Bleached sulfite-dissolved softwood pulp (70% Norway spruce (Picea abeis) and 30% Scots pine (*Pinus sylvestris*)) were received from Domsjö Fabriker AB (Sweden). d,l-lactic acid (90%), sodium hydroxide, and hydrochloric acid (37 wt %) were obtained from VWR BDH chemicals. Ethylene brassylate (1,4-dioxacycloheptadecane-5,17-dione) (>95%) and TBD were purchased from Sigma-Aldrich (Sweden). All chemicals were used as received without further purification.

### Fabrication of Lactic Acid-Functionalized CNF (LACNF)

LACNF was prepared according to the previously reported method.^21^ Briefly, a mixture of bleached sulfite pulp (17.5 g) and acid lactic (90%, 700 mL) was stirred via a mechanical stirrer (1400 rpm) at 105 °C for 24 h. The reaction mixture was cooled to room temperature and centrifuged at 12,000 rpm (14 min) to collect the solid materials. The collected solids were redispersed in water and washed by centrifugation (5 × 700 mL). Next, the washed solid cellulose was dispersed into distilled water (700 mL) and homogenized via IKA T25 ULTRA TURAX at 14,000 rpm for 90 min. To determine the yield of the CNF, the suspension was centrifuged at 12,000 rpm (14 min), and then the water was decanted. The CNF solids were collected and lyophilized. The lyophilized solid CNF was broken down into a powder using a mortar and pestle and dried for 18 h under reduced pressure.

### Hydrolysis of LACNF (CNF)

0.5 g of fabricated LACNF was dispersed in 40 mL of EtOH (aq) 70% v/v, then 30 mL of NaOH (0.5 M) was dropwise added, and the mixture was stirred at 60 °C for 24 h. After cooling down to room temperature, the mixture was centrifugated (12,000 rpm, 14 min), and the solid materials were collected. The collected solids were redispersed in water, washed by centrifugation (3 × 50 mL H_2_O), and finally lyophilized.

### Reactive Extrusion

Before REx, EB was dried overnight in a ventilated oven at 75 °C. The polymerization was carried out with an EB/TBD molar ratio of 42:1 corresponding to 14.84 g of EB (54.9 mmol) and 0.18 g of TBD (1.3 mmol). This ratio resulted in the highest molecular weight among other ratios tested (here not reported for the sake of brevity), confirming results from previous studies.^10^ EB was polymerized neat and in the presence of CNF or LACNF at 1 wt % keeping a constant EB/TBD molar ratio of 42:1. The reaction was performed in an Xplore microcompounder (total volume 15 cm^3^) at 130 °C and 100 rpm with a recirculating system for 60 min under a constant nitrogen flow. Samples of reaction products were withdrawn after 30 min, and the final products were extruded after 60 min. The extrusion force was recorded in line to monitor the reaction. The extruded strands were injection molded with an Xplore IM12 into bars or dumbbell-shaped specimens, with a barrel temperature of 80 °C (mold at 20 °C), following an injection program of 2 s at 280 bar and holding 10 s at 420 bar.

### Mechanical Recycling

The dumbbell-shaped specimens of PEB and the nanocomposites were manually shredded and extruded in a microcompounder (Xplore, total volume of 5 cm^3^) at 130 °C and 100 rpm for 10 min. The extruded strands were injection molded into dumbbell-shaped specimens under the aforementioned conditions. After mechanical and thermal analyses, the specimens were shredded and extruded again other three times, with a total of four recycles, and injection molded. It is worth noting that this mechanical recycling has been designed to simulate postindustrial mechanical recycling.

### Characterization Methods

Soxhlet extraction in chloroform of extruded PEB-CNF was performed for 72 h. The insoluble fraction was dried under reduced pressure for 5 h and tested for structural analysis to determine the possible covalent bonding between PEB and CNF.

Attenuated total reflectance Fourier transformed infrared spectroscopy (ATR-FTIR) was performed within the 400–4000 cm^–1^ wavelength region on a Thermo Scientific NICOLET 6700 FTIR, using OMNIC software, smart orbit ATR diamond crystal 30,000–200 cm^–1^ (110 scans per measurement, and with a resolution of 4 cm^–1^).

The freeze-dried CNF and LACNF were characterized by field emission scanning electron microscopy using a Tescan MAIA3 (voltage 5 kV). Before imaging, the samples were coated with iridium (5 nm) using a Quorum Q150T sputter coater.

The atomic force microscopy (AFM) spectrum was recorded by the NCM method (noncontact mode) using the Park system NX20 instrument.

All transmission electron microscopy (TEM) experiments were carried out on a 200 kV JEOL JEM-2100F field-emission electron microscope equipped with an ultrahigh-resolution pole piece (Cs = 0.5 mm). High-angle annular dark-field images were acquired by a JEOL ADF detector using a Gatan DigitalMicrograph. The incident beam probe was 0.2 nm with a convergence angle of ≈10 mrad, and a camera length of 8 cm was used. The samples were dispersed on a Cu TEM grid with holey carbon supporting films.

Size-exclusion chromatography (SEC) was performed only on PEB, as CNF or LACNF could not be completely filtered out of the nanocomposites. PEB was dissolved in chloroform (CHCl_3_), and SEC was carried out at 30 °C using an Agilent (Diegem, Belgium) liquid chromatograph equipped with an Agilent degasser, an isocratic HPLC pump (flow rate = 1 mL min^–1^), an Agilent autosampler (loop volume = 100 μL; solution concentration = 2 mg mL^–1^), an Agilent-DRI refractive index detector, and three columns: a PL gel 5 μm guard column and two PL gel Mixed-B 5 μm columns (linear columns for the separation of molar mass (PS) ranging from 200 to 4 × 105 g mol^–1^). Polystyrene standards were used for calibration.

Proton nuclear magnetic resonance (^1^H NMR) experiments were carried out in solution-state at 310 K on a Bruker AMX 500 MHz equipped with a 5 mm PABBO BB/19F-1H-D Z-GRD probe. CDCl_3_ was used as a solvent, and 0.3 wt % tetramethylsilane (TMS, 0 ppm) was used as an internal chemical shift reference. Spectra were recorded with a 12.0 ms pulse and 2 s relaxation delay.

The monomer conversion was calculated as the ratio between the integrals of the C*H*_2_O_EB_ (4.31 ppm) and C*H*_2_O_PEB_ (4.27 ppm) signals. The molar mass was calculated as C*H*_2_O_PEB_/(2*C*H*_2_OH_PEB_)*270 + 139, where C*H*_2_O_PEB_ and C*H*_2_OH_PEB_ are the integrals of the signals at 4.27 and 3.83 ppm and 270 and 139 are the molar masses in g mol^–1^ of EB and TBD, respectively. The theoretical molar mass was calculated as [EB]_0_/[TBD]_0_*conversion*270 + 139, where [EB]_0_ and [TBD]_0_ are the initial moles of EB and TBD, thus the ratio being 42.

Solid-state CP/MAS ^13^C NMR spectra were recorded by using a Bruker Avance III 500 MHz spectrometer equipped with a 4 mm HX CP MAS probe. Experiments were acquired at a magic angle spinning (MAS) rate of 10 kHz and a temperature of 298 K. The cross-polarization (CP) experiments used a 90° excitation pulse of 3 ms for 1H, followed by a contact time of 1.5 ms with a ^13^C spin lock frequency of 60 kHz, while 1H was ramped from 45 up to 90 kHz. The 1H decoupling scheme at 83 kHz was applied during the acquisition. The relaxation delay was 2 s, and Adamantane was used as an external reference with the CH_2_ signal at 38.48 ppm.

The thermal transitions of the materials were tested by differential scanning calorimetry (DSC) on a Mettler Toledo DSC 2 calorimeter equipped with a HSS7 sensor and a TC-125MT intercooler. The endotherms were recorded with a heating/cooling/heating profile from −80 to 150 °C at a heating rate of 10 °C min^–1^ under a nitrogen constant flow of 50 mL min^–1^.

The thermal stability was studied by thermogravimetric analysis (TGA) with a Mettler Toledo TGA/DSC 3+ Star system. The samples were preheated from room temperature to 70 °C, where an isothermal segment was maintained for 15 min to remove any absorbed moisture and finally to 500 °C. The heating rate was 5 °C min^–1^ under a nitrogen constant flow of 50 mL min^–1^.

Dynamic mechanical thermal analysis (DMTA) was carried out with a DMA Q800 (TA Instruments) apparatus in tension-film mode on rectangular bars (25 × 5 × 1 mm^3^). The bars were cut from injection molded films and conditioned for 48 h at 23 °C and 53% relative humidity. Tanδ, storage, and loss moduli were recorded during frequency sweeps between 0.1 and 50 Hz at a strain of 0.1% and a preload force of 0.01 N at 25 °C. Temperature sweeps between −45 and 45 °C were also performed at 1 Hz and 0.1% strain.

Tensile properties were measured on dumbbell-shaped specimens with a gauge length of 25 mm and a thickness of around 2 mm, conditioned for 48 h at 23 °C and 53% relative humidity. A Zwick/Z2.5 tensile tester equipped with a 2 kN load cell was used at a test speed of 2.5 mm min^–1^ (10% deformation rate) according to the standard ASTM D638–14.

The injection-molded samples were cryofractured in liquid nitrogen, and the surfaces were gold-sputtered with an Edwards Sputter Coater S150B at 1.2 kV and 15 mA for 30 s. The surfaces were investigated with a Zeiss Sigma Ultra 55 FEG-SEM instrument under a 5 kV accelerating voltage.

## Results and Discussion

### CNF Extraction and Functionalization

**Figure 1:**
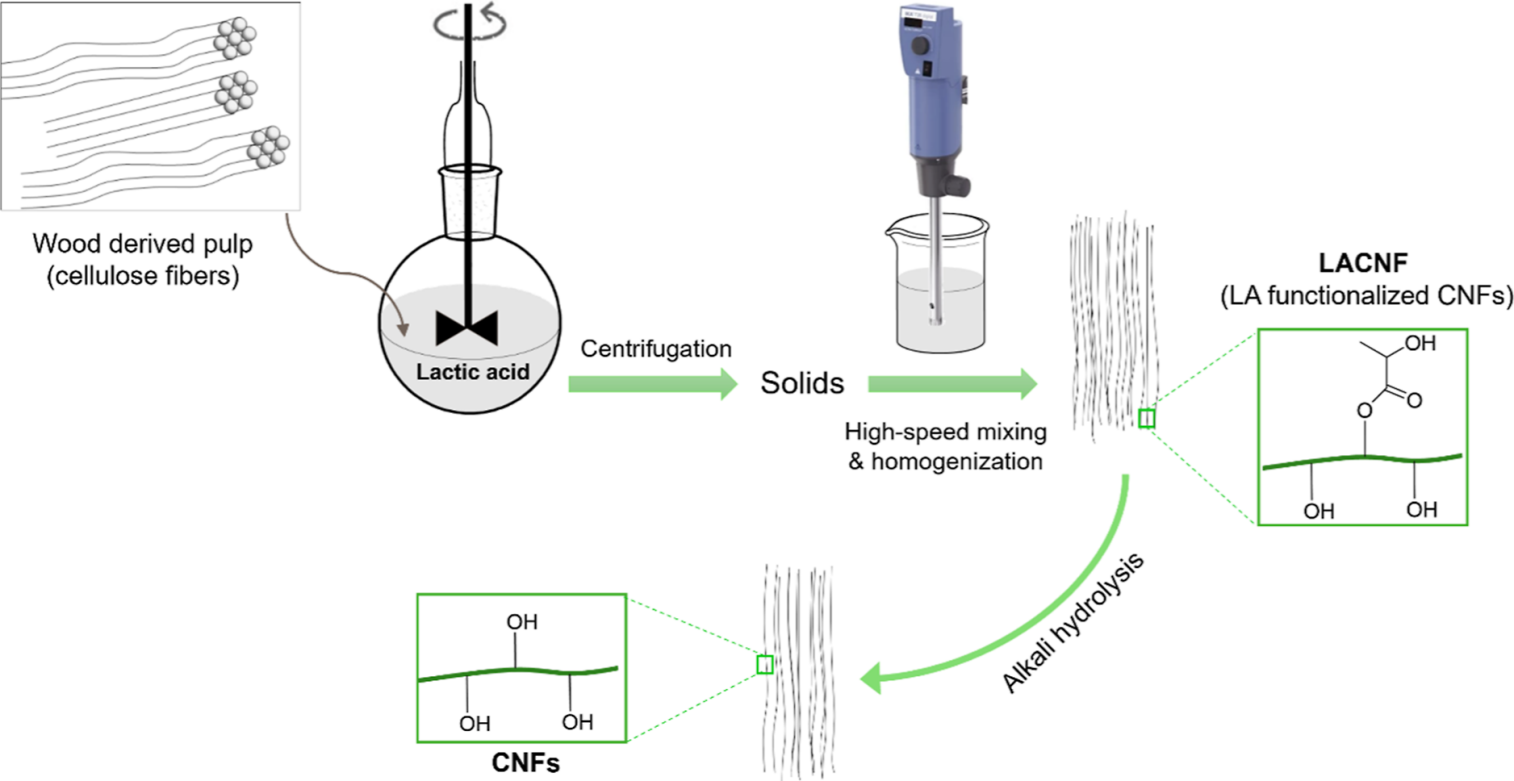
Scheme illustrating the fabrication of lactic acid-esterified nanofibrils (LACNF) and their alkali hydrolysis to produce cellulose nanofibrils.

LACNF and CNF were successfully fabricated (Figure 1) via a novel lactic acid autocatalyzed and concurrent esterification process.^21^ Briefly, lactic acid was used as both a reaction media and catalyst to fabricate LACNF from wood-derived bleached sulfite pulp. The method is a one-step reaction performed in neat reaction media without the use of toxic substances or metal-based catalysts. LACNF had a degree of substitution of 0.2 measured by alkaline titration and nanostructure with an aspect ratio ≈162 ± 24.^21^

**Figure 2:**
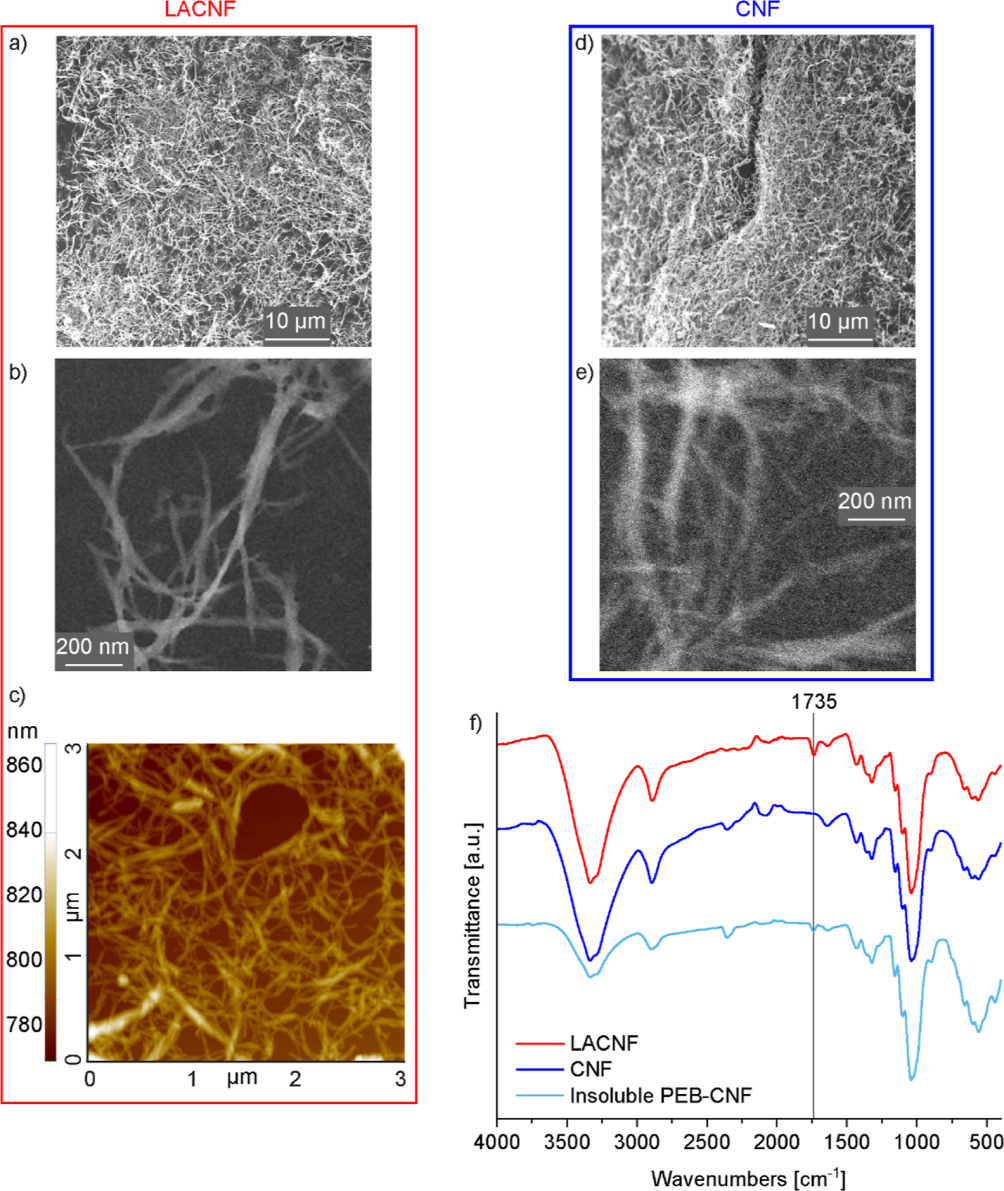
Scanning electron micrographs of freeze-dried (a) lactic acid-esterified nanofibrils (LACNF) and (d) CNF. TEM of (b) LACNF and (e) CNF. (c) AFM of LACNF. (f) ATR-FTIR spectra of LACNF, CNF, and PEB-CNF after Soxhlet extraction with CNF as the initiator.

The esterification is reversible; therefore, the ester groups of LACNF have been removed using alkaline conditions for the production of unmodified cellulose nanofibrils (CNF), with unchanged morphology (Figure 2). The spectrum recorded by ATR-FTIR of LACNF (Figure 2f) shows the characteristic peak of the ester bond at around 1737 cm^–1^, confirming that the lactic acid esterification of the hydroxyl groups of the CNF. On the other hand, ATR-FTIR analysis of the CNF shows that the ester bond peak has disappeared (Figure 2f). The aspect ratio of CNF was almost unchanged compared to the LACNF, with an average length of around 1230 ± 220 nm and diameter of 8 ± 2 nm measured on around 100 different individualized CNF.

### ROP via REx

**Figure 3:**
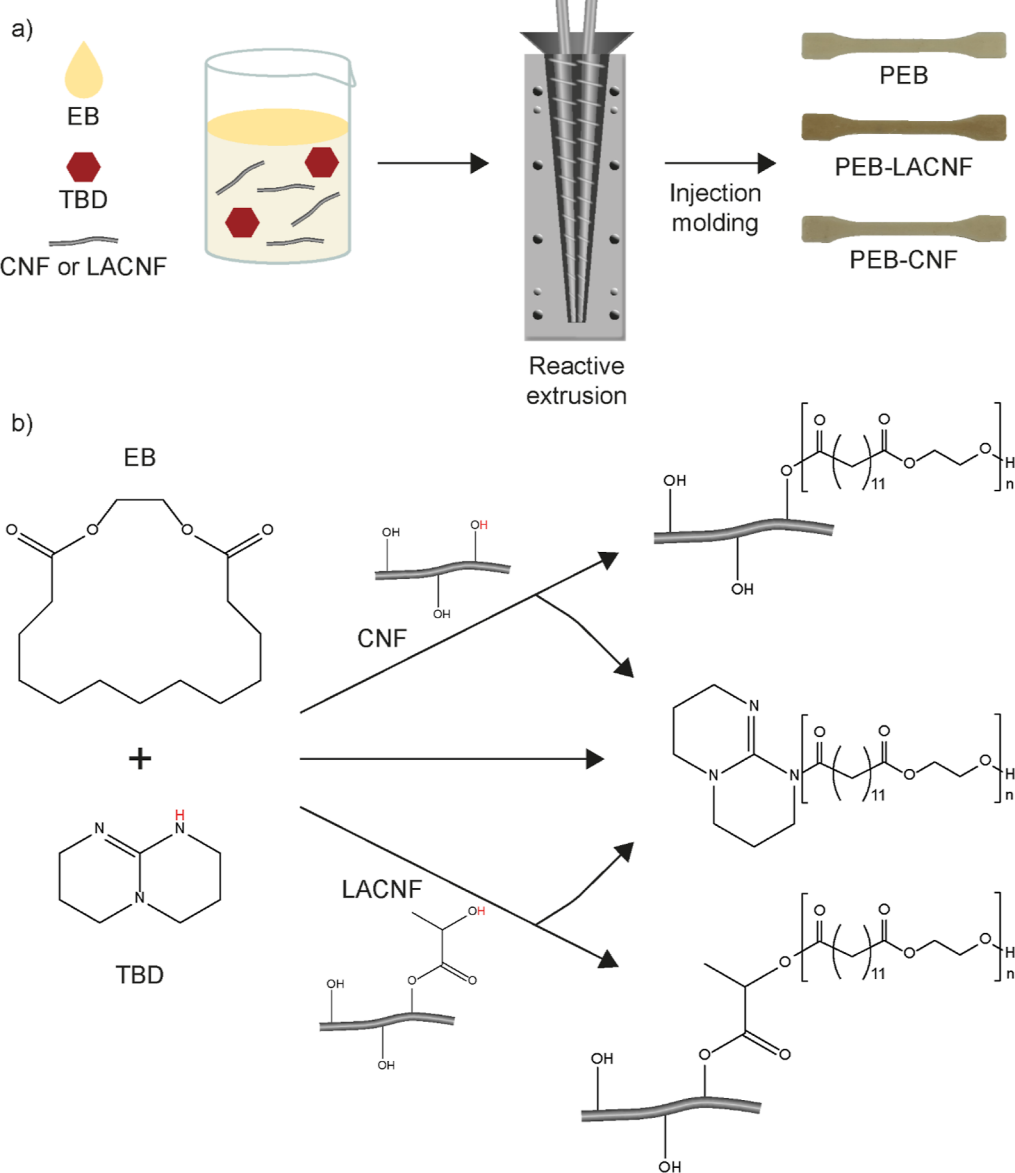
(a) Scheme of materials fabrication through REx and photo of injection-molded tensile specimens. (b) Scheme of reaction between EB and TBD in the presence of CNF or lactic acid-esterified nanofibrils (LACNF).

The ROP of EB was carried out as a one-step REx in a microcompounder, which is a sustainable method that does not require solvents and can omit the need for purification, as illustrated in Figure 3a. The ROP was mediated by organic TBD (EB/TBD 42:1 molar ratio), which has the bifunctional role of catalyst and initiator.^11,12^ The reaction was also carried out in the presence of 1 wt % CNF or LACNF to generate in situ and simultaneously manufacture nanocomposites in a single-step process, to evaluate whether the CNF topochemistry influences the polymerization and the nanocomposites’ properties. The possibility of grafting PEB from the CNF hydroxyl groups was investigated as a strategy to promote CNF dispersion and interaction with PEB. The chemical structures of the expected products are schematized in Figure 3b. The reaction was monitored via an in-line recording of the extrusion force, which is proportional to the viscosity of the system under the processing conditions (Figure S1). The force increased steadily up to 30 min from the REx start, when it dropped in connection to the withdrawal of extrudate samples for further testing and finally reached a plateau at about 40 min. The in-line measurement of the extrusion force is a function of the mass in the extruder. Therefore, a withdrawal of the mass at 30 min reduced the force needed for compounding at a constant speed (100 rpm). The processing could be continued for up to 60 min with no substantial deflection of the force. The increase in the force during the first 40 min indicated an increase in the viscosity of the system due to polymerization and a related increase in molar mass within this time gap, while the steady process (plateau) during an additional 20 min allowed the completion of monomer conversion without further significant molar mass increase and attested for the polymer stability, i.e., no polyester degradation occurred under these processing conditions.^15^ The force, i.e., the viscosity, of the CNF-based system showed a slightly higher slope, which could imply different polymerization kinetics for the two systems. It is worth noting that carbonyl moieties could lead to transesterification reactions during the synthesis of biodegradable polyesters, therefore competing with ROP.^22^ Transesterification is a second-order reaction and takes place during the polymerization.^23,24^ Due to the lactate ester moieties on LACNF surfaces, transesterification reactions might occur during PEB growing, slowing down the polymerization kinetics. Moreover, thermomechanical degradation favors hydrolysis of lactate moieties to lactic acid, which in turns catalyzes the degradation of PEB during melt processing, counteracting its growth.^25^ Both of these hypotheses could explain the slower increase of the extrusion force.

The molar masses and monomer conversion were measured by SEC and ^1^H NMR. Both the samples were withdrawn at 30 min, and the final products at 60 min of REx were tested as representative systems to evaluate how the reaction progressed during the REx processing. The characterizations were carried out in chloroform in which the polyester was soluble and the nanocomposites were dispersible. SEC analysis was not performed on the nanocomposites because it was not possible to filter out the CNF or LACNF from the nanocomposite dispersions. The nanocomposites were only characterized by solution ^1^H NMR, under the hypothesis that the soluble fraction could also represent the PEB fraction eventually grafted from CNF and therefore accounted for in the insoluble fraction. This hypothesis assumes that the ROP kinetic would not be affected by the initiator, which could be TBD, CNF or LACNF, or water molecules eventually present. Hence, the achieved PEB molar mass would be the same regardless of the initiator. Moreover, in the solid-state NMR spectrum of the insoluble fraction, commented below, the end-groups of PEB are not resolvable from the noise of the signal to enable an accurate calculation of the molar mass.

The number (*M*_nSEC_) and weight (*M*_wSEC_) average molar masses of neat PEB are 13 and 40 kDa, respectively, after 30 min of Rex (Table 1). These values slightly decrease after 60 min with an increase of polydispersity (*D̵*) from 2.9 to 3.1. Large *D̵* values are reported for ROP carried out in Rex and can be the consequence of transesterification reactions, which may increase with the processing time.^26^

**Table 1:** Molar Masses, Polydispersity, and Monomer Conversion of PEB and the Nanocomposites Tested at 30 and 60 min Rex Time

<!—Col Count:7F0E0material	*M*_nSEC_^a^ [kDa]	*M*_wSEC_^a^[kDa]	*D̵*^a^	conversion^b^ [%]	*M*_nNMR_^b^[kDa]	*M*_ntheor_^c^ [kDa]
PEB_t30	13	40	2.9	94	8.1	10.8
PEB_t60	12	36	3.1	94	8.1	10.7
PEB-LACNF_t30	n.a.	n.a.	n.a.	98	9.2	11.3
PEB-LACNF_t60	n.a.	n.a.	n.a.	99	9.3	11.3
PEB-CNF_t30	n.a.	n.a.	n.a.	98	9.0	11.3
PEB-CNF_t60	n.a.	n.a.	n.a.	99	10.0	11.3

a From SEC.

b From ^1^H NMR.

c The theoretical molar mass was calculated as [EB]_0_/[TBD]_0_ × conversion × 270 + 139, where [EB]_0_ and [TBD]_0_ are the initial moles of EB and TBD, thus the ratio being 42.

As previously mentioned, when the PEB synthesis was performed in the presence of CNF and LACNF, the nanofibrils could not be separated from the dispersions by filtration, and no sedimentation of nanofibrils was observed. The difficulty of separating the nanofibrils indicates their good dispersion, most probably in an individualized nanostructure, which suggests their surface modification with PEB, i.e., at an effective grafting from the nanofibril surface hydroxyl moieties. This hypothesis was verified by extensive Soxhlet extraction of the PEB-CNF nanocomposite, which removed all the noncovalently bonded PEB from the insoluble fraction, before its structural characterization (ATR-FTIR and solid-state CP/MAS ^13^C NMR). The only effective way to prove the grafting of PEB onto CNF (or LACNF) was to investigate the presence of ester bonds. Therefore, both FTIR and solid-state NMR were performed only on the insoluble fraction extracted from PEB-CNF. Because LACNF already contains esters due to the surface modification of the nanofibrils, the analyses on the insoluble fraction of PEB-LACNF would not be conclusive on the PEB grafting as the signal of PEB grafted on LACNF would overlap with the signal of the lactate moieties.

**Figure 4:**
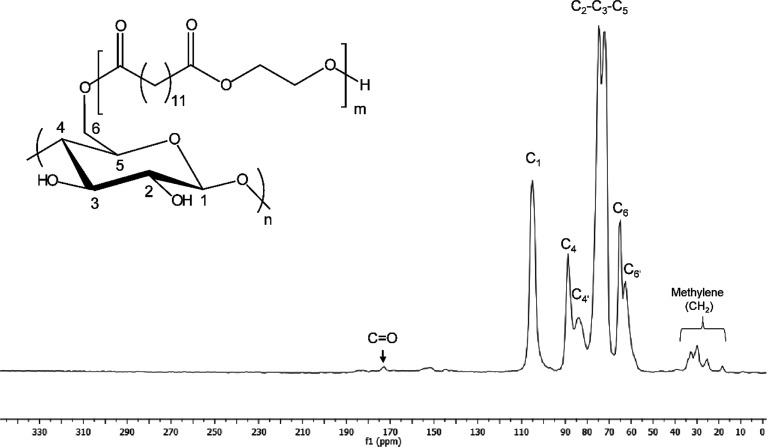
Solid-state CP/MAS ^13^C NMR spectrum of PEB-CNF with the schematic assignment of specific resonance peaks detected in the spectrum.^21^

ATR-FTIR of the Soxhlet-extracted CNF revealed the presence of ester bonds with a peak at around 1735 cm^–1^ (Figure 2f). This observation confirms the covalent bonding of PEB onto CNF, proving the grafting. Furthermore, solid-state CP/MAS ^13^C NMR was performed on the insoluble fraction recovered from the CNF-based nanocomposite. The spectrum of the insoluble fraction from PEB-CNF (Figure 4) depicts the characteristic carbon resonances of cellulose at around 105, 89, and 65 ppm for C1, C4, and C6, respectively. C4′ and C6′ are assigned to amorphous parts that come at 84 and 63 ppm, while C4 and C6 are assigned to the ordered cellulose structure. The cluster at 72–75 ppm belongs to C2–C3–C5 in cellulose.^21^ However, in the spectrum of CNF, a peak at 174 ppm is noticeable and can be ascribed only to the carbonyl group (CO) and peaks between 18 and 32 ppm belong to the methylene (CH_2_) groups of PEB, confirming the covalent bonding of PEB onto the CNFs. It is worth noting that the choice of performing ATR-FTIR and solid-state CP/MAS ^13^C NMR only on the CNF-based system and not on the LACNF intended to prevent a misleading interpretation of lactate moieties’ resonance peaks (between 18 and 21 ppm)^27,28^ with peaks belonging to the methylene (CH_2_) groups of PEB (overlapping in the region of 18–32 ppm).

**Figure 5:**
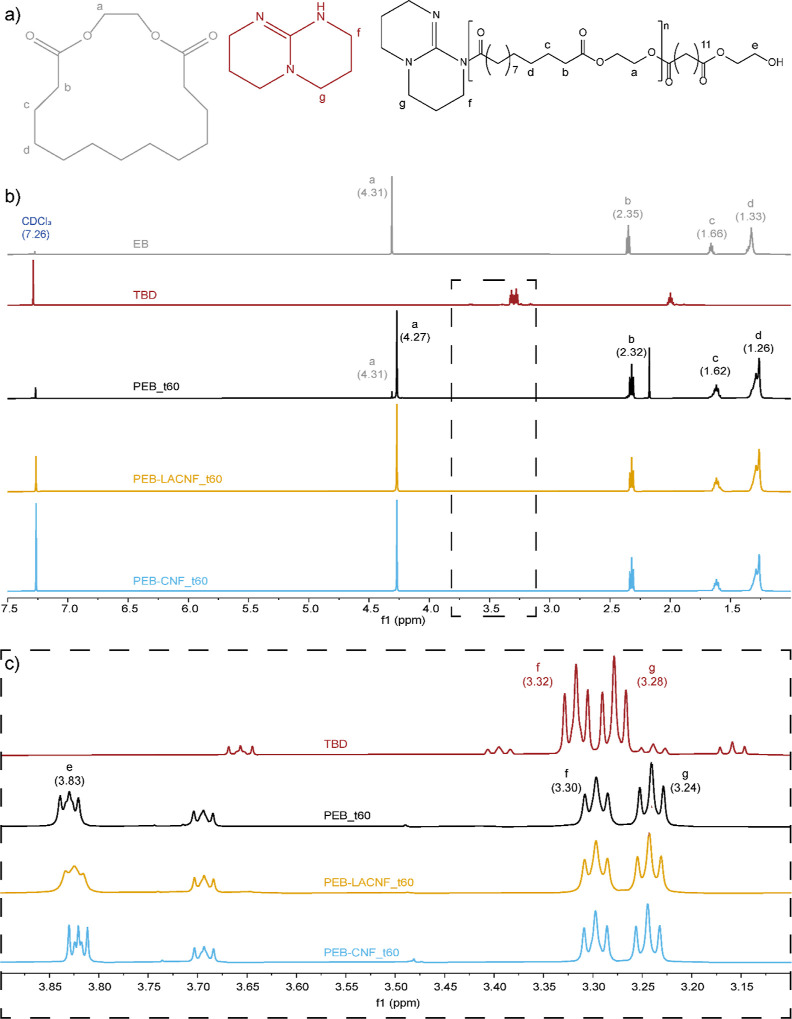
(a) EB, TBD, and PEB structures. (b) ^1^H NMR spectra in CDCl_3_ at room temperature of EB, TBD, PEB, and the nanocomposites after 60 min REx and (c) inset between 3.10 and 3.90 ppm. The signal assignment is reported with the parts per million shift in parentheses.

The ^1^H NMR spectra in CDCl_3_ of PEB and the nanocomposites are similar, showing signals related to the protons in the polymer chains (4.27, 2.32, 1.62, and 1.26 ppm) and the C*H*_2_O in the monomer (4.31 ppm) with minor intensity (Figure 5). In the spectra, there are two triplets attributed to the TBD (3.30 and 3.24 ppm), which are shifted compared to the spectrum of free TBD (3.32 and 3.28 ppm), confirming its contribution as a ROP initiator.^11,12^ The monomer conversion calculated by ^1^H NMR was above 90% for all the systems after 30 min of REx with no significant difference after 60 min (Table 1), confirming the feasibility of EB polymerization via REx and suggesting that the reaction could be stopped after 30 min. The molar mass of the nanocomposites calculated by ^1^H NMR (*M*_nNMR_) was ≈ 9 kDa, with no significant differences between the two nanocomposites (differences around 7% of the *M*_nNMR_, in the limit of the integration accuracy). The calculation was based on the ratio between the integrals of the C*H*_2_O signal at 4.27 ppm (a) and C*H*_2_OH at 3.83 ppm (e).^11^

The theoretical molar mass (*M*_ntheor_) ≈ 11 kDa was calculated assuming that all of the TBD acts as the initiator, intentionally neglecting the initiation from nanofibrils. The analysis of the results led to values of *M*_nNMR_ lower than the *M*_ntheor_, in line with the structural analysis demonstrating that the hydroxyl and lactic acid moieties on the nanofibrils are also active sites for the initiation of the ROP and compete with the initiation from TBD. In this case, the TBD still plays a catalyst role leading to higher conversion,^11^ and its interaction with cellulose cannot be excluded, as free TBD is not detected in the ^1^H NMR spectra.

The results demonstrate that REx is a feasible single-step method for the ROP of EB and the production of in situ CNFs/PEB nanocomposites.^10,12^

### Nanocomposites Properties

The thermal analyses (DSC and TGA) were performed to assess the possible effects of the CNF or LACNF on the thermal transitions and the thermal degradation of PEB. According to DSC, PEB shows a typical semicrystalline behavior, and it is characterized by multiple melting endotherms between 30 and 75 °C, with the maximum at 71 °C, indicating a heterogeneous crystal population, in agreement with its polydispersity (Table 2 and Figure S2). The melting enthalpy (Δ*H*_m_) of PEB is 88 J g^–1^, in accordance with the value reported in the literature for similar molar mass.^29^ The presence of CNF slightly increases Δ*H*_m_, indicating a slight nucleating effect, while it does not relevantly affect the melting (*T*_m_) or crystallization (*T*_c_) temperatures. In agreement with the high melting enthalpies, no *T*_g_ was visible in DSC. From TGA, CNF slightly increases (by 2 °C) the onset of degradation of PEB, while LACNF decreases it by 8 °C (Table 2 and Figure S3). During the polymerization, transesterification might occur between growing PEB and the LACNF lactate esters moieties, thus liberating lactic acid which catalyzes the polyester thermal degradation.^25,28,30^ However, these small variations of the onset temperature of degradation can be ascribed to the slight differences in molecular weight of the nanocomposites compared to neat PEB.

**Table 2:** Thermal Properties of PEB and the Nanocomposites^a^

material	*T*_g_ [°C]^1^	*T*_m_ [°C]^2^	Δ*H*_m_ [J g^–1^]^2^	*T*_c_ [°C]^3^	*T*_5%_ [°C]^4^	*T*_d_ [°C]^5^
PEB	–19	71	88.3	56	376	439
PEB-LACNF	–17	72	92.5	55	368	438
PEB-CNF	–14	72	94.2	55	378	441

a The glass transition temperature (*T*_g_) corresponds to the loss modulus peak of the DMTA temperature sweep. The melting temperature (*T*_m_) corresponds to the maximum peak of melting in the DSC second heating, and its enthalpy (Δ*H*_m_) is integrated between 30 and 75 °C. The crystallization peak (*T*_c_) is evaluated in the DSC cooling step. The temperature at 5% weight loss (*T*_5%_) has been assessed by TGA. The peak temperature of degradation (*T*_d_) was measured from the TGA first derivative.

**Figure 6:**
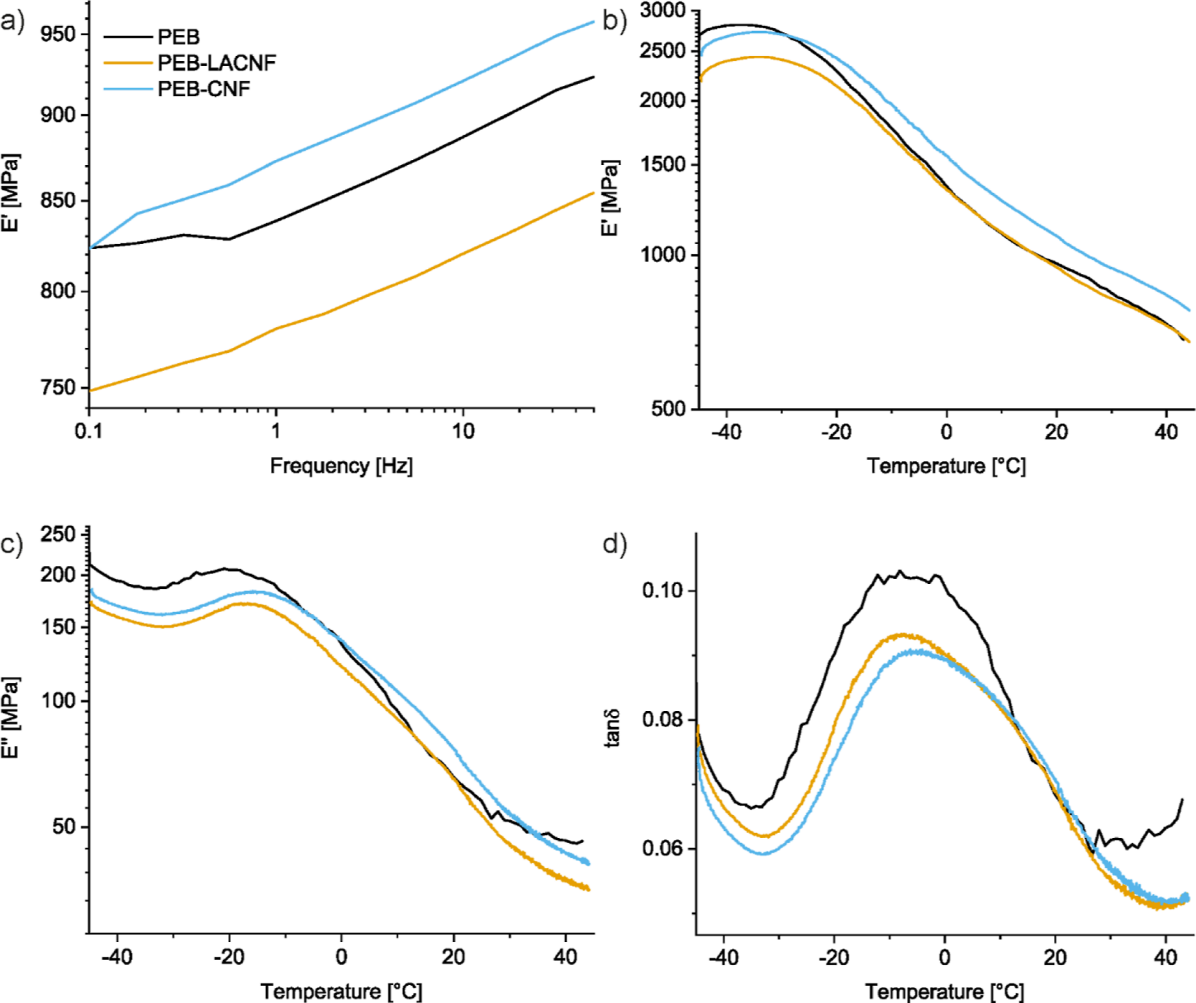
Representative dynamic mechanical thermal analysis curves of PEB and the in situ PEB nanocomposites: storage moduli (*E*′) recorded in (a) frequency sweep at 25 °C and (b) in temperature sweep at 1 Hz; (c) loss moduli (*E*″) and (d) tan δ recorded in temperature sweep.

As *T*_g_ could not be detected by DSC, DMTA in tensile mode was used to assess both the thermal transitions and the dynamic mechanical behavior of the materials. The loss modulus in the temperature sweep shows a peak at −19 °C for PEB, related to its glass transition (Table 2 and Figure 6). This peak is shifted to a higher temperature when the polymerization is carried out in the presence of LACNF or CNF, supporting a molecular interaction between the rigid nanocellulose and the polyester that limits the PEB macromolecular chains’ mobility, thus delaying its glass transition. Unmodified CNF led to higher *T*_g_, indicating a greater interaction between PEB and CNF than with LACNF. The shift of the *T*_g_ to a higher temperature with only 1 wt % nanofibrils demonstrates their fine dispersion, confirming the in situ approach as a valuable method to obtain nanocomposites preserving nanofibrils’ individualization. Such change was not observed in PEB-CNC nanocomposites produced by Burton et al.^3^ in which the *T*_g_ was unchanged even at 50 wt % CNC. To discriminate the interfaces of the two nanofibril types with PEB, further investigation on nanoscale features would be beneficial. However, when low *T*_g_ matrices are involved, as in PEB, artifacts are generated during sample preparation of thin slices and flat surfaces for tests such as TEM or AFM, making the testing not conclusive. The storage modulus recorded in both frequency (at 25 °C) and temperature sweeps (at 1 Hz) shows that PEB-CNF is the most elastic material above the *T*_g_ (Figure 6). PEB-CNF is also characterized by the smallest damping factor (tan δ), a further indication of improved elastic character and greater interaction between the polyester matrix and the CNF. The lower values of PEB-LACNF moduli registered both in the frequency and temperature sweeps are in line with possible degradation phenomena induced by the lactate moieties and consistent with the TGA.

The mechanical properties were further evaluated by tensile tests at room temperature (Table 3 and Figure S4). PEB exhibits a Young’s modulus of ≈300 MPa, larger than poly(ε-caprolactone) with significantly higher molar mass,^31^ but it fractures at low elongations (≈3%). The stiff and brittle behavior is a consequence of the relatively low molecular weight achieved, being not large enough to allow sufficient chain entanglement, therefore hindering high deformations.^32^ The Young’s modulus is increased by LACNF and CNF by 42 and 46%, respectively, compared to neat PEB. An increase in the elongation at break of 59 and 47% compared to neat PEB is shown for the LACNF and CNF nanocomposites, respectively. These results can be attributed both to the reinforcement effect provided by the nanofibrils and to the increment in the molar mass of the in situ polymerized PEB in the presence of the nanofibrils. It should be noted that the differences among the average tensile values of the nanocomposites are not significant because within the scattering of the data. Therefore, there is no difference among the mechanical properties of the two nanocomposites, and both nanofibrils reinforce the PEB. This is in agreement with the similar molar masses of the nanocomposites measured by ^1^H NMR (Table 1). Moreover, due to the low mass fraction of the nanofibrils, a high level of dispersion can be hypothesized as a reason for the improvement of tensile properties.

**Table 3:** Tensile Properties with Standard Deviations of PEB and the Nanocomposites Measured at Room Temperature

materials	Young’s modulus [MPa]	tensile strength [MPa]	elongation at break [%]
PEB	247 ± 10	8.9 ± 2.6	3.4 ± 1.0
PEB-LACNF	352 ± 6	13.7 ± 1.3	5.4 ± 0.4
PEB-CNF	362 ± 18	12.8 ± 0.7	5.0 ± 0.6

**Figure 7:**
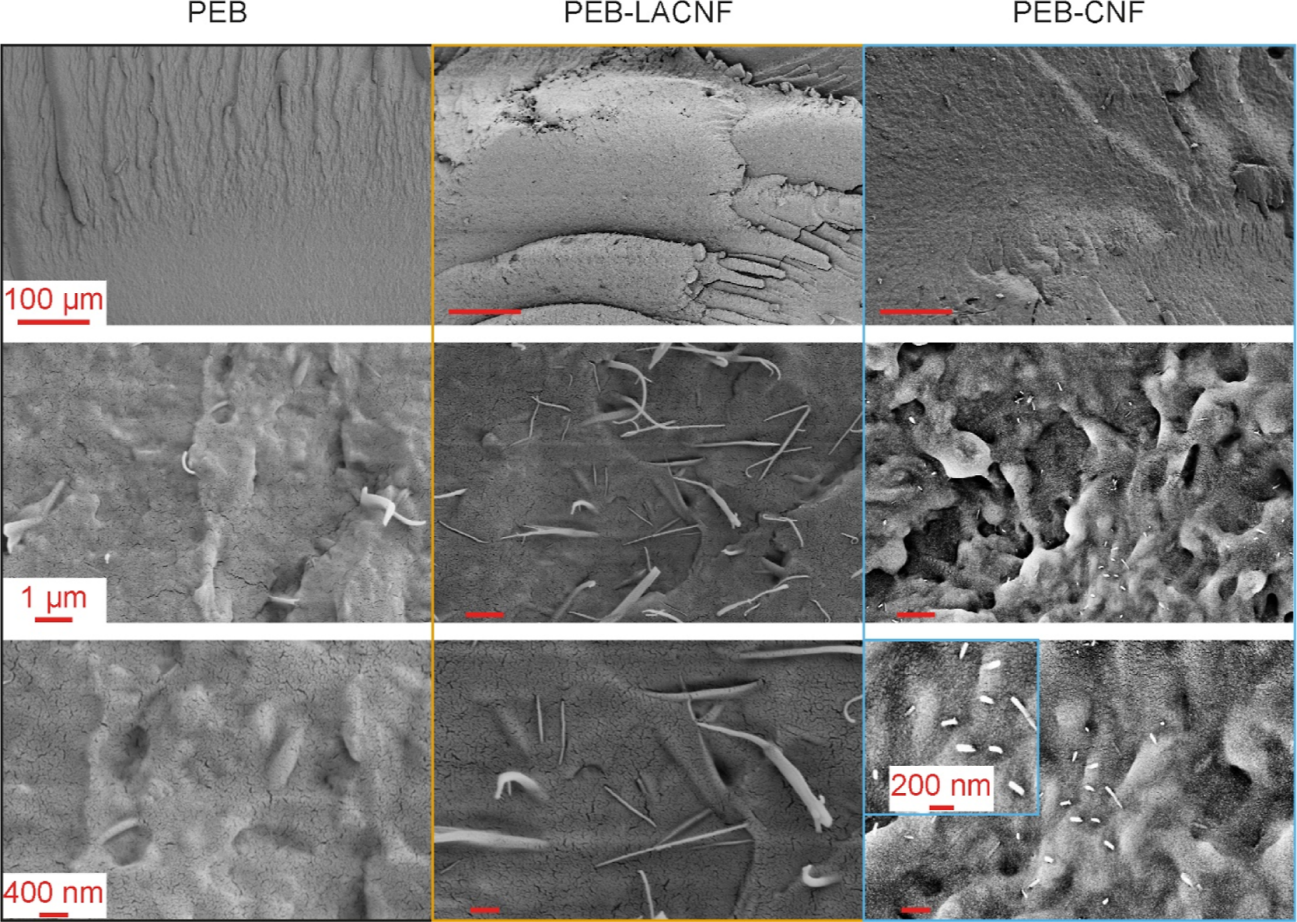
Micrographs of fractured surfaces of PEB and the in situ PEB-CNF or LACNF nanocomposites obtained by scanning electron microscopy at different magnifications with scale bars at 100 μm, 1 μm, and 400 nm. An inset at higher magnification with a 200 nm scale bar is reported for PEB-CNF (bottom right image).

The fracture surfaces of the materials were analyzed by SEM to investigate the nanofibrils’ dispersion and interaction with PEB. All the materials show rough brittle fractures (Figure 7). At the magnification with a 1 μm scale bar, elongated polymer fibrils are present on the surface of PEB and PEB-LACNF. In both nanocomposites, nanoscopic inclusions emerge, which can be ascribed to CNF or nanosized CNF bundles, with a diameter of ≈40 nm similar to the initial dimensions of the freeze-dried bundles of CNF used for the in situ polymerization in REx (Figure 2). It is worth noting that the direction of the cryofractured surfaces could not be controlled due to the brittleness of samples, resulting in nanofibrils oriented in different directions for the two nanocomposites (LACNF directed more parallel, while CNF was more perpendicular to the cryofracture surfaces). The inclusions are only part of the nanofibrils, while the rest is embedded in the PEB matrix; therefore, the length observed is not representative of the entire nanofibrils. The thicknesses of LACNF and CNF are, instead, representative and similar. Both the CNF and LACNF appear well dispersed in the polymer matrix and have good adhesion with PEB, as a consequence of the in situ polymerization and *grafting from* polymerization. At the different magnifications studied, no pull-outs or debonding are detectable in the morphology of the nanocomposites, regardless of the different topochemistry.

**Figure 8:**
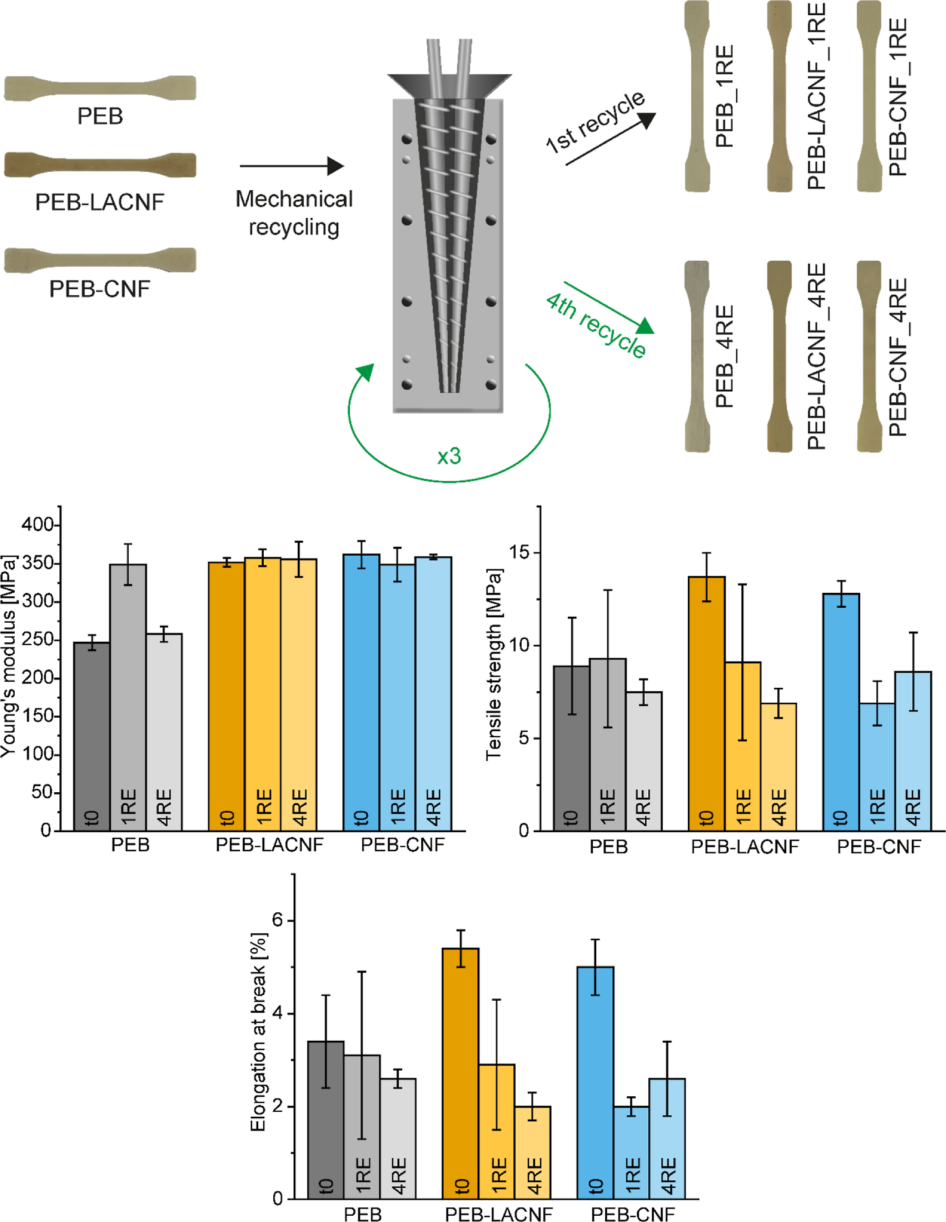
Scheme illustrating the mechanical recycling with the injection molding of tensile specimens after the first and fourth recycles. Young’s modulus, tensile strength, and elongation at break measured by tensile tests of the materials as produced (*t*0), after the first (1RE) and fourth (4RE) recycles.

The feasibility of mechanical recycling was evaluated as a possible end-of-life solution for the nanocomposites together with the impact of recycling on the material properties. The injection-molded specimens were shredded and reprocessed four times to simulate postindustrial mechanical recycling via extrusion. Tensile bars were injected after the first and fourth cycles to test the mechanical properties (Figure 8). The results show that the stiffness of the materials was not significantly affected by mechanical recycling. The tensile strength and elongation of the nanocomposites are halved after four recycles, reaching values similar to those of PEB. Embrittlement indirectly confirms a decrease in molar mass caused by degradation during the repeated extrusion. The thermal analyses (Figures S5 and S6) show a detrimental effect of four recycles especially on PEB-LACNF, characterized by a reduction of PEB melting temperatures and increase of crystallization temperature and by a significant reduction of the onset of degradation evaluated by TGA. These results are in line with a molar mass decrease induced by lactic acid-catalyzed degradation during extrusion.^28,30^ The mechanical recycling of PEB nanocomposites is therefore possible; however, it further enhances the brittleness of the materials.

## Conclusions

This work explored a solvent-free method for organo-mediated polymerization of EB and in situ fabrication of PEB/CNF or lactate-modified (LACNF) nanocomposites. EB, a renewable and relatively cheap macrolactone, was polymerized in the presence of CNF or LACNF via REx using TBD as the catalyst. The nanofibrils were extracted via a lactic-acid-mediated esterification process, which is autocatalytic. The lactate moieties were removed from the fabricated LACNF surface by alkaline hydrolysis, yielding unmodified CNF while preserving the pristine aspect ratio. PEB and the nanocomposites fabricated via REx exhibit a molar mass of about 9 kDa. The extruded materials were successfully injection molded into bars and dumbbell-shaped specimens, confirming good melt processability in conventional melt processing equipment. The analyses indicated that the materials were stiff and brittle and that unmodified CNF and LACNF increased the deformability of PEB by 47 and 59% and its elasticity by 47 and 42%, respectively. The morphology of the nanocomposites revealed an effective dispersion and adhesion of the nanofibrils to the polymer matrix, confirming the in situ polymerization as a valuable method to preserve nanofibril individualization during melt extrusion. This work demonstrates the polymerization of EB by REx, a green and fast method that can be easily scaled up. This method can open for industrial production of PEB and its cellulose nanocomposites with the potential introduction on the market of novel biobased, biodegradable, and mechanically recyclable materials. Further advancement of the reaction conditions and catalysts would tune the PEB properties and performance, e.g., its molecular weight. A range of properties can facilitate the industrial scaleup and the development of PEB-based (nano)composites for various applications, targeting the replacement of fossil-based nonbiodegradable thermoplastics.

## Supporting Information Available

Force recorded during extrusion of the nanocomposites; second heating and cooling of DSC; TGA and DTG; representative tensile curves; second heating and cooling of DSC of the recycled materials; and TGA of the recycled materials (PDF)

## References

[ref1] Rosenboom J. G.; Langer R.; Traverso G. Bioplastics for a Circular Economy. Nat. Rev. Mater. 2022, 7 (2), 117–137. 10.1038/s41578-021-00407-8. 35075395 PMC8771173

[ref2] Müller S.; Uyama H.; Kobayashi S. Lipase-Catalyzed Ring-Opening Polymerization of Cyclic Diesters. Chem. Lett. 1999, 28 (12), 1317–1318. 10.1246/cl.1999.1317.

[ref3] Butron A.; Llorente O.; Fernandez J.; Meaurio E.; Sarasua J. R. Morphology and Mechanical Properties of Poly(Ethylene Brassylate)/Cellulose Nanocrystal Composites. Carbohydr. Polym. 2019, 221 (June), 137–145. 10.1016/j.carbpol.2019.05.091. 31227152

[ref4] Fernández J.; Amestoy H.; Sardon H.; Aguirre M.; Varga A. L.; Sarasua J. R. Effect of Molecular Weight on the Physical Properties of Poly(Ethylene Brassylate) Homopolymers. J. Mech. Behav. Biomed. Mater. 2016, 64, 209–219. 10.1016/j.jmbbm.2016.07.031. 27517665

[ref5] He M.; Cheng Y.; Liang Y.; Xia M.; Leng X.; Wang Y.; Wei Z.; Zhang W.; Li Y. Amino Acid Complexes with Tin as a New Class of Catalysts with High Reactivity and Low Toxicity towards Biocompatible Aliphatic Polyesters. Polym. J. 2020, 52 (6), 567–574. 10.1038/s41428-020-0314-0.

[ref6] Jin C.; Wei Z.; Yu Y.; Sui M.; Leng X.; Li Y. Copolymerization of Ethylene Brassylate with δ-Valerolactone towards Isodimorphic Random Copolyesters with Continuously Tunable Mechanical Properties. Eur. Polym. J. 2018, 102, 90–100. 10.1016/j.eurpolymj.2018.03.018.

[ref7] Li J.; Wang S.; Lu H.; Tu Y.; Wan X.; Li X.; Tu Y.; Li C. Y. Helical Crystals in Aliphatic Copolyesters: From Chiral Amplification to Mechanical Property Enhancement. ACS Macro Lett. 2023, 12 (3), 369–375. 10.1021/acsmacrolett.2c00753. 36847524

[ref8] Wang X.; Wang X.; Zhen N.; Gu J.; Zhang H.; Dong B.; Wang F.; Liu H. Sodium Complexes Bearing Cavity-like Conformations: A Highly Active and Well-Controlled Catalytic System for Macrolactone Homo- and Copolymerization. Polym. Chem. 2021, 12 (13), 1957–1966. 10.1039/D0PY01580F.

[ref9] Wei Z.; Jin C.; Xu Q.; Leng X.; Wang Y.; Li Y. Synthesis, Microstructure and Mechanical Properties of Partially Biobased Biodegradable Poly (Ethylene Brassylate- Co - ε -Caprolactone) Copolyesters. J. Mech. Behav. Biomed. Mater. 2019, 91 (December 2018), 255–265. 10.1016/j.jmbbm.2018.12.019. 30599448

[ref10] Pascual A.; Sardon H.; Veloso A.; Ruipérez F.; Mecerreyes D. Organocatalyzed Synthesis of Aliphatic Polyesters from Ethylene Brassylate: A Cheap and Renewable Macrolactone. ACS Macro Lett. 2014, 3 (9), 849–853. 10.1021/mz500401u. 35596349

[ref11] Pascual A.; Sardón H.; Ruipérez F.; Gracia R.; Sudam P.; Veloso A.; Mecerreyes D. Experimental and Computational Studies of Ring-Opening Polymerization of Ethylene Brassylate Macrolactone and Copolymerization with ε-Caprolactone and TBD-Guanidine Organic Catalyst. J. Polym. Sci., Part A: Polym. Chem. 2015, 53 (4), 552–561. 10.1002/pola.27473.

[ref12] Kim S.; Chung H. Synthesis and Characterization of Lignin-Graft-Poly(Ethylene Brassylate): A Biomass-Based Polyester with High Mechanical Properties. ACS Sustain. Chem. Eng. 2021, 9 (44), 14766–14776. 10.1021/acssuschemeng.1c04334.

[ref13] Fritz-Langhals E. Unique Superbase TBD (1,5,7-Triazabicyclo[4.4.0]Dec-5-Ene): From Catalytic Activity and One-Pot Synthesis to Broader Application in Industrial Chemistry. Org. Process Res. Dev. 2022, 26 (11), 3015–3023. 10.1021/acs.oprd.2c00248.

[ref14] Ottou W. N.; Sardon H.; Mecerreyes D.; Vignolle J.; Taton D. Update and Challenges in Organo-Mediated Polymerization Reactions. Prog. Polym. Sci. 2016, 56, 64–115. 10.1016/j.progpolymsci.2015.12.001.

[ref15] Spinella S.; Ganesh M.; Lo Re G.; Zhang S.; Raquez J. M.; Dubois P.; Gross R. A. Enzymatic Reactive Extrusion: Moving towards Continuous Enzyme-Catalysed Polyester Polymerisation and Processing. Green Chem. 2015, 17 (8), 4146–4150. 10.1039/C5GC00992H.

[ref16] Eichhorn S. J.; Dufresne A.; Aranguren M.; Marcovich N. E.; Capadona J. R.; Rowan S. J.; Weder C.; Thielemans W.; Roman M.; Renneckar S.; Gindl W.; Veigel S.; Keckes J.; Yano H.; Abe K.; Nogi M.; Nakagaito A. N.; Mangalam A.; Simonsen J.; Benight A. S.; Bismarck A.; Berglund L. A.; Peijs T. Review: Current International Research into Cellulose Nanofibres and Nanocomposites. J. Mater. Sci. 2010, 45 (1), 1–33. 10.1007/s10853-009-3874-0.

[ref17] Carlmark A.; Larsson E.; Malmström E. Grafting of Cellulose by Ring-Opening Polymerisation - A Review. Eur. Polym. J. 2012, 48 (10), 1646–1659. 10.1016/j.eurpolymj.2012.06.013.

[ref18] Hafrén J.; Córdova A. Direct Organocatalytic Polymerization from Cellulose Fibers. Macromol. Rapid Commun. 2005, 26 (2), 82–86. 10.1002/marc.200400470.

[ref19] Córdova A.; Hafrén J. Direct Organic Acid-Catalyzed Polyester Derivatization of Lignocellulosic Material. Nord. Pulp Pap. Res. J. 2005, 20 (4), 477–480. 10.3183/npprj-2005-20-04-p477-480.

[ref20] Casas J.; Persson P. V.; Iversen T.; Córdova A. Direct Organocatalytic Ring-Opening Polymerizations of Lactones. Adv. Synth. Catal. 2004, 346 (9–10), 1087–1089. 10.1002/adsc.200404082.

[ref21] Rafi A. A.; Alimohammadzadeh R.; Avella A.; Mõistlik T.; Jűrisoo M.; Kaaver A.; Tai C. W.; Lo Re G.; Cordova A. A Facile Route for Concurrent Fabrication and Surface Selective Functionalization of Cellulose Nanofibers by Lactic Acid Mediated Catalysis. Sci. Rep. 2023, 13 (1), 14730 10.1038/s41598-023-41989-3. 37679445 PMC10484996

[ref22] Lipik V. T.; Widjaja L. K.; Liow S. S.; Abadie M. J. M.; Venkatraman S. S. Effects of Transesterification and Degradation on Properties and Structure of Polycaprolactone-Polylactide Copolymers. Polym. Degrad. Stab. 2010, 95 (12), 2596–2602. 10.1016/j.polymdegradstab.2010.07.027.

[ref23] Collins S.; Kenwright A. M.; Pawson C.; Peace S. K.; Richards R. W.; MacDonald W. A.; Mills P. Transesterification in Mixtures of Poly(Ethylene Terephthalate) and Poly(Ethylene Naphthalene-2,6-Dicarboxylate): An NMR Study of Kinetics and End Group Effects. Macromolecules 2000, 33 (8), 2974–2980. 10.1021/ma9916382.

[ref24] Zhu Z.; Xiong C.; Zhang L.; Deng X. Synthesis and Characterization of Poly(?-Caprolactone)-Poly(Ethylene Glycol) Block Copolymer. J. Polym. Sci., Part A: Polym. Chem. 1997, 35 (4), 709–714. 10.1002/(SICI)1099-0518(199703)35:4<709::AID-POLA14>3.0.CO;2-R.

[ref25] Carrasco F.; Pagès P.; Gámez-Pérez J.; Santana O. O.; Maspoch M. L. Processing of Poly(Lactic Acid): Characterization of Chemical Structure, Thermal Stability and Mechanical Properties. Polym. Degrad. Stab. 2010, 95 (2), 116–125. 10.1016/j.polymdegradstab.2009.11.045.

[ref26] Liu P.; Wu J.; Yang G.; Shao H. Comparison of Static Mixing Reaction and Reactive Extrusion Technique for Ring-Opening Polymerization of L-Lactide. Mater. Lett. 2017, 186 (September 2016), 372–374. 10.1016/j.matlet.2016.10.024.

[ref27] Magnani C.; Idström A.; Nordstierna L.; Müller A. J.; Dubois P.; Raquez J. M.; Lo Re G. Interphase Design of Cellulose Nanocrystals/Poly(Hydroxybutyrate- Ran-Valerate) Bionanocomposites for Mechanical and Thermal Properties Tuning. Biomacromolecules 2020, 21 (5), 1892–1901. 10.1021/acs.biomac.9b01760. 32078304

[ref28] Spinella S.; Lo Re G.; Liu B.; Dorgan J.; Habibi Y.; Leclère P.; Raquez J. M.; Dubois P.; Gross R. A. Polylactide/Cellulose Nanocrystal Nanocomposites: Efficient Routes for Nanofiber Modification and Effects of Nanofiber Chemistry on PLA Reinforcement. Polymer 2015, 65, 9–17. 10.1016/j.polymer.2015.02.048.

[ref29] Marxsen S. F.; Song D.; Zhang X.; Flores I.; Fernández J.; Sarasua J. R.; Müller A. J.; Alamo R. G. Crystallization Rate Minima of Poly(Ethylene Brassylate) at Temperatures Transitioning between Quantized Crystal Thicknesses. Macromolecules 2022, 55, 3958–3973. 10.1021/acs.macromol.2c00308.

[ref30] Román-Ramírez L. A.; McKeown P.; Shah C.; Abraham J.; Jones M. D.; Wood J. Chemical Degradation of End-of-Life Poly(Lactic Acid) into Methyl Lactate by a Zn(II) Complex. Ind. Eng. Chem. Res. 2020, 59 (24), 11149–11156. 10.1021/acs.iecr.0c01122. 32581423 PMC7304880

[ref31] Avella A.; Mincheva R.; Raquez J.-M.; Lo Re G. Substantial Effect of Water on Radical Melt Crosslinking and Rheological Properties of Poly(ε-Caprolactone). Polymers 2021, 13 (4), 491–505. 10.3390/polym13040491. 33557338 PMC7915490

[ref32] Hill A.; Ronan W. Relationship between Failure Strain, Molecular Weight, and Chain Extensibility in Biodegradable Polymers. J. Mech. Behav. Biomed. Mater. 2023, 139 (October 2022), 105663 10.1016/j.jmbbm.2023.105663. 36657195

