# Supplementary material for: Organo-Mediated Ring-Opening Polymerization of Ethylene Brassylate from Cellulose Nanofibrils in Reactive Extrusion
Source: ACS Sustain Chem Eng. 2024 Jul 12;12(29):10727–38. doi: 10.1021/acssuschemeng.4c01309 (PMC11267636; doi:10.1021/acssuschemeng.4c01309)
Supplement: Supplementary file 1 — sc4c01309_si_001.pdf [file sc4c01309_si_001.pdf]

Supporting Information

Organo-mediated ring-opening polymerization of ethylene brassylate from cellulose nanofibrils in reactive extrusion

Angelica Avella†, Abdolrahim Rafi‡, Luca Deiana‡, Rosica Mincheva§, Armando Córdova‡, Giada Lo Re†**

†Department of Industrial and Materials Science, Chalmers University of Technology, Rännvägen 2A, Göteborg, 41258, Sweden.

‡Department of Natural Sciences, Mid Sweden University, Holmgatan 10, Sundsvall, 85170, Sweden.

§Laboratory of Polymeric and Composite Materials (LPCM), Center of Innovation and Research in Materials and Polymers (CIRMAP), University of Mons, Mons, 7000, Belgium.

Corresponding Authors

*Giada Lo Re giadal@chalmers.se +46 31 772 64 80; Armando Córdova armando.cordova@miun.se

This supplemental document contains 7 pages (S1-S7) with Figures S1-S6.

Contents:

Figure S1: Force recorded during extrusion of the nanocomposites ……………….S2

Figure S2: Second heating and cooling of DSC ……………………………………….S3

Figure S3: TGA and DTG of PEB and the nanocomposites………..………………..S4

Figure S4: Representative tensile curves ……………………...……………………....S5

Figure S5: Second heating and cooling of DSC of the recycled materials…………S6

Figure S6: TGA of the recycled materials ……………………………………………...S7

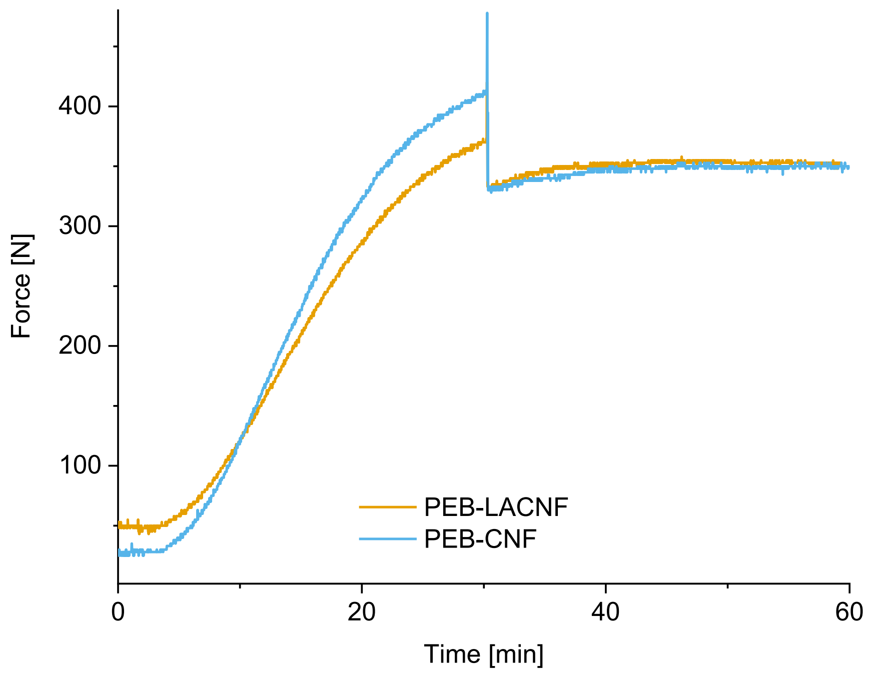

Figure S1. Extrusion force recorded in line during reactive extrusion of poly(ethylene brassylate) polymerized with 1 wt.% LACNF or 1 wt.% CNF. The drop of force at 30 min reaction time is due to samples withdrawal.

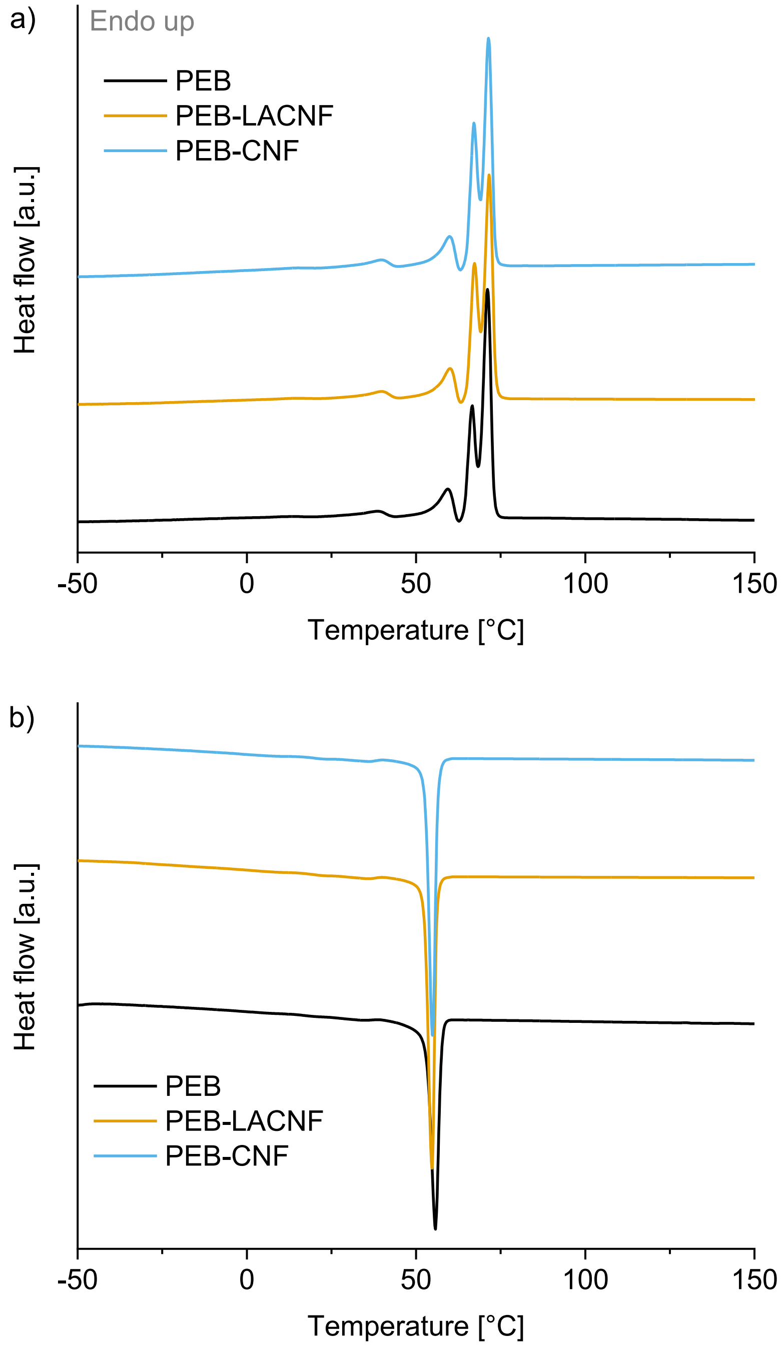

Figure S2. a) Second heating scan of differential scanning calorimetry of poly(ethylene brassylate) and the nanocomposites and b) cooling scan.

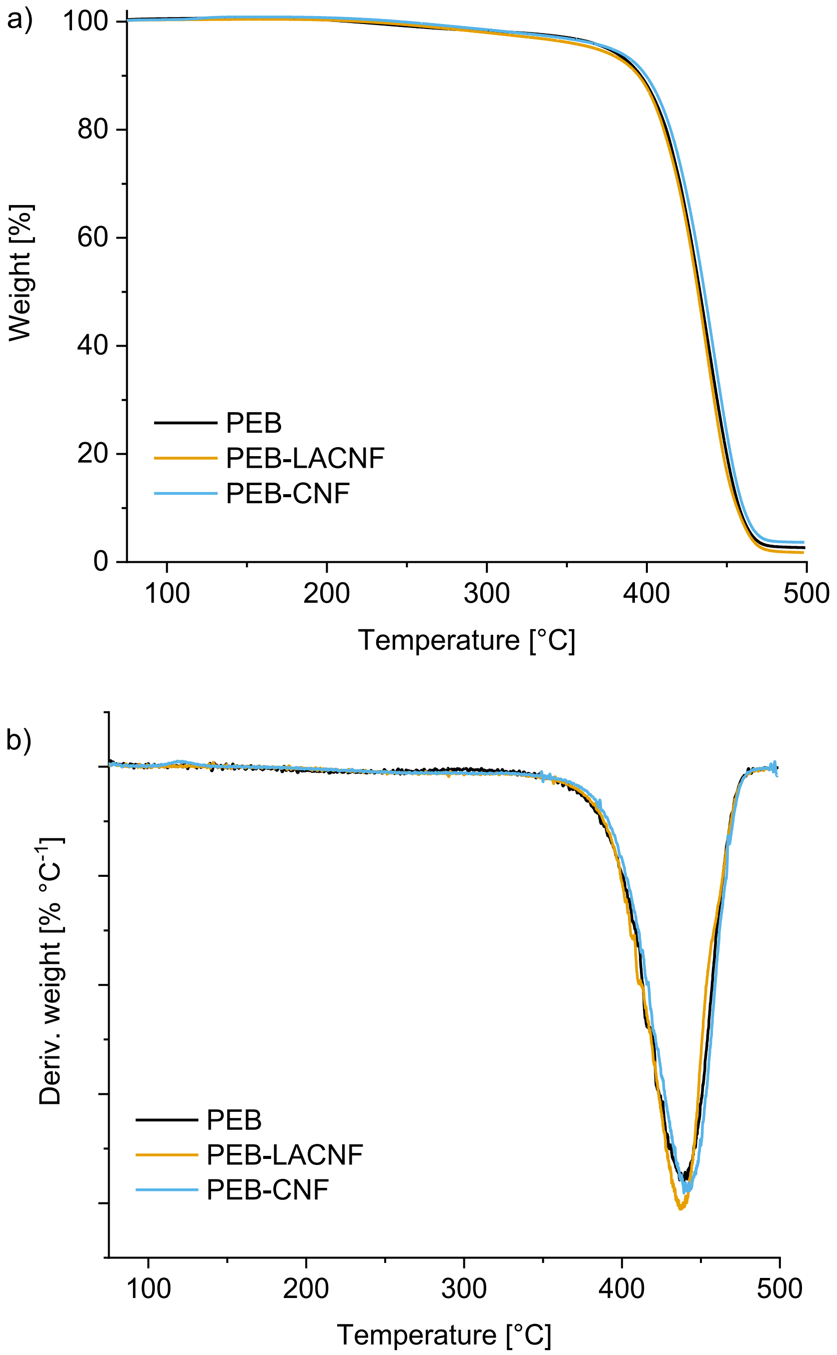

Figure S3. a) Thermogravimetric analysis in nitrogen of poly(ethylene brassylate) and the nanocomposites and b) its first derivative.

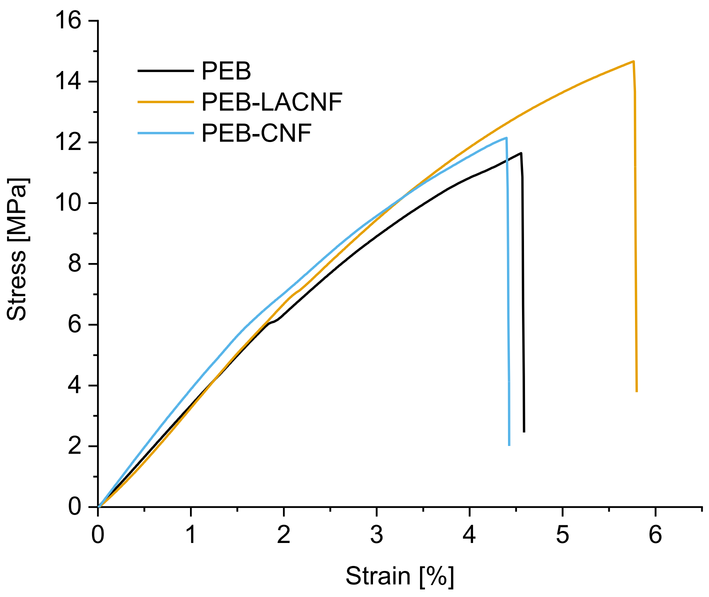

Figure S4. Representative tensile curves of poly(ethylene brassylate) and the nanocomposites tested at room temperature on injection moulded dog bone-shaped specimens.

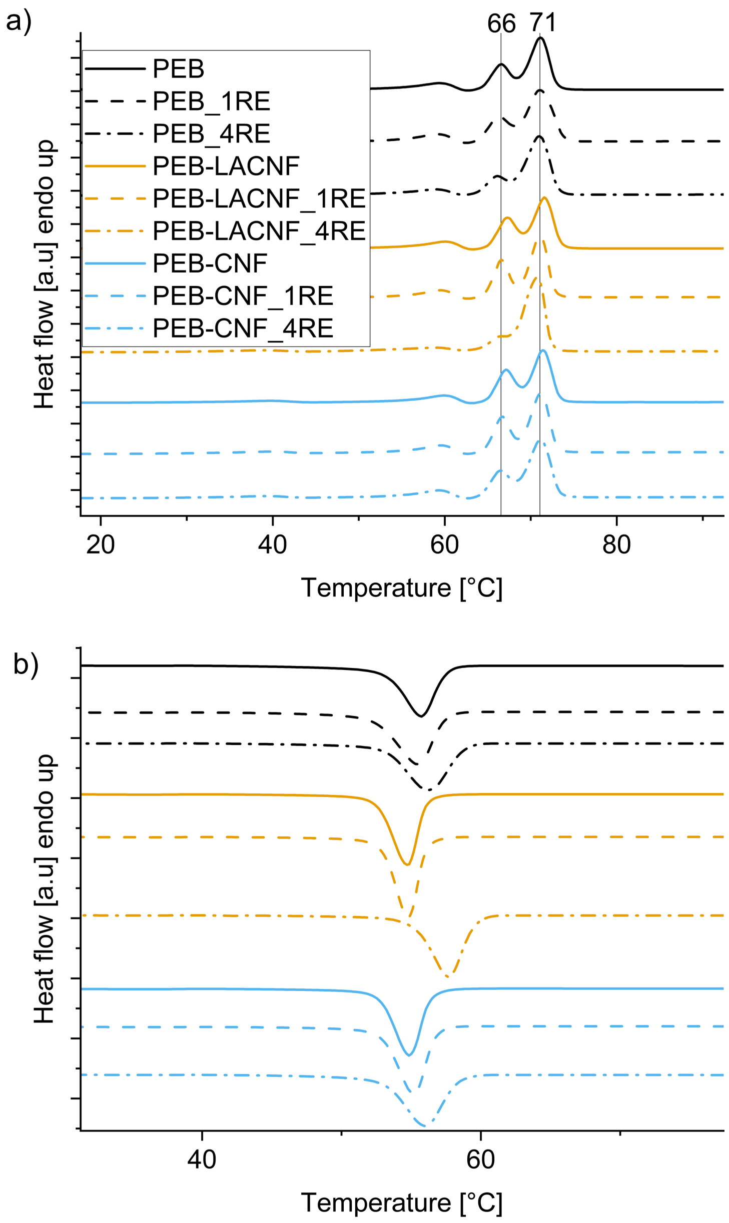

Figure S5. a) Second heating scan and b) cooling scan of differential scanning calorimetry of poly(ethylene brassylate) and the nanocomposites as processed and after one (1RE) and four (4RE) recycles.

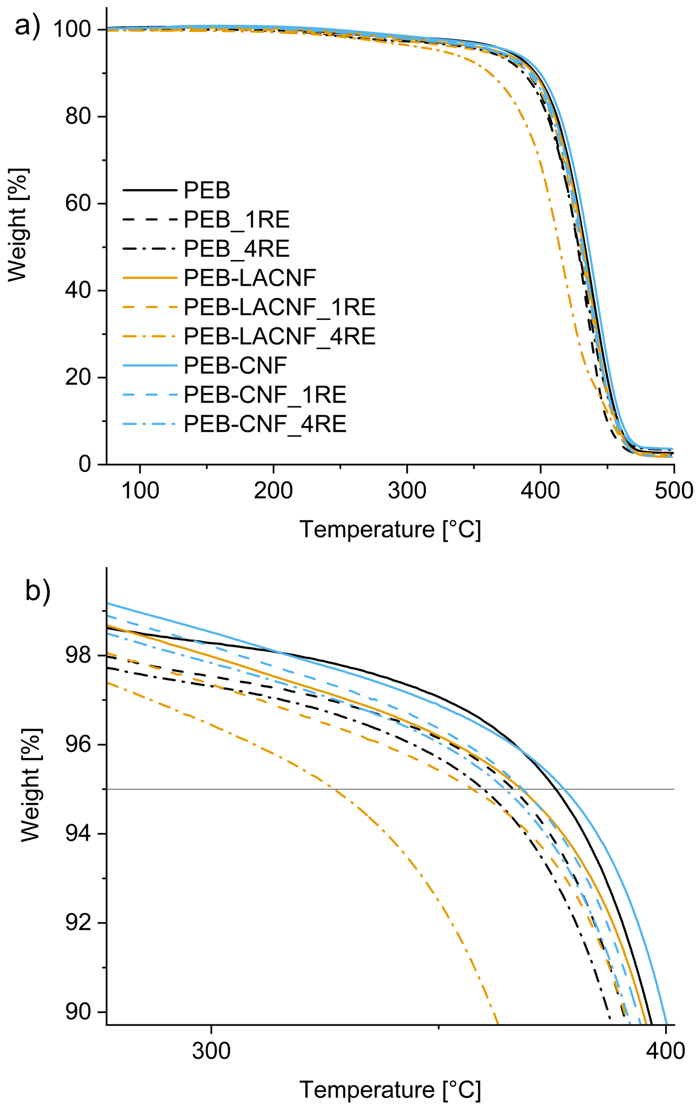

Figure S6. a) Thermogravimetric analysis in nitrogen and b) zoom on the onset of degradation of poly(ethylene brassylate) and the nanocomposites as processed and after one (1RE) and four (4RE) recycles.
